# Attention to the Values, Wishes and Needs of Patients With Advanced Cancer by Hospital Clinicians, an Exploratory Qualitative Study

**DOI:** 10.1177/10499091241261025

**Published:** 2024-06-18

**Authors:** Sita de Vries, Laury Pijnappel, Sigrid Vervoort, Yvette van der Linden, Saskia Teunissen, Everlien de Graaf

**Affiliations:** 1 Center of Expertise in Palliative Care, Julius Center for Health Sciences and Primary Care, 8124 University Medical Center Utrecht, Utrecht, The Netherlands; 2 General practice and Nursing Science, Julius Center for Health Sciences and Primary Care, 8124 University Medical Center Utrecht, Utrecht, The Netherlands; 3 The Netherlands Comprehensive Cancer Organisation, Utrecht, The Netherlands; 4 Center of Expertise in Palliative Care, 4501 Leiden University Medical Center, Leiden, The Netherlands

**Keywords:** palliative care, communication, patient-centered care, hospitals, quality of life, qualitative research

## Abstract

**Context:** Insight into patients’ personal values, wishes, and needs (VWN) by clinicians is essential to guide appropriate palliative care. **Objective:** To gain insight into the exploration and monitoring of the VWN of patients with advanced cancer during the illness trajectory by hospital oncology clinicians. **Method:** A generic qualitative study was conducted from February 2022 to July 2022. Specialized nurses, nurse practitioners and medical specialists (in training) providing care to adult patients with advanced cancer were recruited at an outpatient clinic in a Dutch academic hospital. Data were collected using in-depth semi-structured interviews and participatory observations of outpatient clinic consultations. Data were analyzed collaboratively by two researchers using thematic analysis. **Results:** Eleven clinicians, aged 33-64, 8 females, participated; 7 interviews and 13 observations were conducted. How clinicians explored and monitored patients’ VWN was based on their opinions, originating from the clinicians’ personal values and work experiences. These were influenced by the local collaboration. Three key opinions were identified: (1) providing safety, (2) supporting medical decision-making, and (3) ensuring alignment. Individual clinicians’ approaches varied. The alignment of care and treatment with the patient’s VWN was observed to be limited. **Conclusion:** Clinicians acknowledged the importance of exploring and monitoring patients’ VWN but lacked a systematic approach in discussing these topics. Patients should be actively engaged in communication regarding their VWN rather than primarily being provided with medical information. Patient-Reported Outcome Measures may be beneficial in facilitating communication regarding the patient’s VWN and could improve appropriate palliative care in hospital cancer care.

## Introduction

Early and systematic integration of palliative care in cancer care improves the patient’s quality of life.^1^ Palliative care aims to optimize the quality of life for patients with life-limiting illnesses through the relief and prevention of multidimensional suffering caused by physical symptoms, psychological issues, social problems, and/or existential needs.^2^ An essential building block is the alignment of care with the patient’s values, wishes and needs (VWN).^3–5^

Effective communication between the patients and clinicians is crucial to guide appropriate palliative care.^6^ First by getting to know the person behind the illness by insight into the patient’s values: what means the most in life to a person, and what living well means to them.^3,7^ Furthermore is discussion of the patient’s multidimensional wishes and needs required.^3^ It is essential that VWN not be discussed once but receive ongoing attention throughout the illness trajectory, as patients’ VWN may change when the illness progresses, requiring palliative care to be adapted appropriately.^8^

Hospital clinicians appear to struggle in discussing the patient’s VWN.^9^ They perceive several barriers e.g. prognostic uncertainty, fear of the impact and feeling inadequately trained.^10^ Patients often wait until a topic is addressed by their clinician, whereas clinicians rely on patients to initiate the conversation, resulting in an ongoing cycle of non-discussion that could affect the patients’ quality of life.^6^ However, it is the clinician’s responsibility to address patients’ VWN and to empower them to share what matters to them.^11^

Further insight into the attention paid to patients’ VWN during the illness trajectory from a clinician’s perspective is required. This study therefore aims to shed light on exploring and monitoring patients’ VWN throughout the illness trajectory by hospital clinicians engaged in oncological care. This insight will support future research in improving the attention to the patients’ VWN to optimize appropriate palliative care in hospitals.

## Method

### Design

A generic qualitative study was conducted to gain insight into exploring and monitoring patients’ VWN by clinicians in a hospital setting.^12^ In-depth semi-structured interviews and participatory observations were used to gain a comprehensive understanding of what clinicians consider important and how this is reflected in clinical practice. The COnsolidated criteria for REporting Qualitative studies (COREQ) were followed.^13^

### Population and Setting

The purposive sample consisted of hospital clinicians providing inpatient and outpatient care to adult patients with advanced cancer. Eligible clinicians were: (1) nurses, nurse practitioners, physicians, or clinicians in training who (2) provide inpatient and outpatient care to adult patients diagnosed with advanced cancer. In addition, outpatient clinic consultations with patients suffering from advanced cancer were selected. These patients either lacked curative treatment options or followed an proactive integrated care pathway in which treatment with both curative and palliative intentions was combined.^14^

Clinicians were recruited at an outpatient clinic of a Dutch academic hospital. A convenience sample was employed, wherein clinicians were included voluntarily based on their own availability and suitable observation moments.^15^ To ensure an adequate range of variation in the clinician’s characteristics, including profession, gender, age and work experience, some clinicians were specifically invited to participate.^16^

Thirteen eligible clinicians were approached by the researcher (LP) face-to-face during weekly multi-professional consultation meetings. They were informed about the study’s purpose and what their participation would involve. If they agreed to participate, interviews and observations were scheduled.

### Study Outcome

The study outcome was the discussion of patients’ VWN by hospital clinicians throughout the illness trajectory, identified as exploring and monitoring, defined as follows:

- Exploring: clinicians gain insight into the person beyond the illness by discussing values: what matters most in life, definitions of living well and consideration of multidimensional wishes and needs.^17–19^
- Monitoring: clinicians consistently focus on patients’ VWN and engage in discussions with patients about their VWN as the illness progresses, recognizing that these may alter as the illness advances.^8^

### Data Collection

Semi-structured interviews and participatory observations were alternated. Participants could be interviewed once and/or observed multiple times. All data were collected by an experienced nurse and nursing scientist in training (LP) between February 2022 and July 2022. No relationship existed between the researcher and the participants before this study started. The interviews started with the question: “*When talking about exploring and monitoring patients’ VWN, can you tell me what this means to you?*” Subsequently, the topic guide^6,20^ was used to delve into how clinicians explore and monitor the patient’s VWN (Appendix 1). Interviews were audio-recorded. The observations were structured using an observation-framework^21,22^ (Appendix 2). The researcher (LP) assumed the role of a complete observer, abstaining from participation in the consultation while seated on the side of the room. Participants were informed that observations aimed to map patient-clinician communication without specifying communication about VWN, to avoid influencing the findings by raising awareness. After each observation, the researcher initiated a discussion with the participant to inquire about the participant’s actions and intentions during the consultation. The observations and subsequent discussions were elaborated into field notes. The topic guide and observation framework were developed based on existing literature and the expertise of the research group.^6,20–22^ Two pilot interviews and two pilot observations were conducted to train the researcher and optimize both forms. Data were collected until the analysis did not reveal any new themes and the existing themes were confirmed.^23^

### Data Analysis

Data were analyzed using thematic analysis^24,25^: (1) interviews were transcribed verbatim and observation notes were elaborated in field notes, both in Dutch. These were read and reread to gain familiarity with the data. (2) Initial codes were noted. (3) Codes were gathered into potential themes. (4) Themes were further refined. (5) Detailed descriptions were provided, identifying the “essence” and naming of themes. Subsequently, themes were translated into English. (6) The final analysis represented the data in relation to the aim and literature. Finally, the quotes provided in this manuscript were translated into English. Two researchers, LP and SdV, who has advanced experience in qualitative research, analyzed the data collaboratively. Codes, themes, and translations were compared frequently. Differences in interpretation were discussed to achieve consensus. A senior researcher (EdG) was consulted if consensus was not reached. NVivo12 supported the analysis.^26^

### Trustworthiness

Mtethod triangulation was applied combining interviews and observations, to obtain a broader understanding of the exploration and monitoring of VWN and to increase credibility.^27^ Investigator triangulation was used to enhance confirmability.^27^ Member checks were applied by summarizing and verifying participant’s responses during interviews and after observations to correct errors in the researchers’ interpretations.^28^ Reflexivity was promoted by memos that recorded reflections on the researchers’ role and personal assumptions.^16,29^ Furthermore, findings and English translations were deliberated during monthly meetings of the research group (SdV, LP, SV, ST, EdG) to enhance confirmability.^30^

### Ethical Issues

The study was conducted following the principles of the Declaration of Helsinki^31^ and the General Data Protection Regulation.^32^ The Medical Research Ethics Committee Utrecht assessed that the Medical Research Involving Human Subjects Act did not apply to this study (21-848/C, January 2022). An independent quality officer of the University Medical Center Utrecht approved the quality of the study protocol. Patients and their loved ones granted verbal consent for the researcher’s attendance during consultations. The refusal of one patient resulted in the researcher not attending this consultation. To ensure confidentiality; data were pseudonymized and department details were omitted.^33^

## Results

### Participant Characteristics

Thirteen eligible clinicians were approached, two of whom declined participation due to time constraints or unspecified reasons. In total, eleven clinicians working in oncology care, specializing in either medical or surgical oncology, participated. Among them were eight women and three men, with a mean age of 52.5 years (range 33-64) (Table 1). Seven interviews and thirteen observations were conducted. Three participants were both interviewed and observed. The interviews lasted 38 minutes on average (range 31-48 min) and were held at the participant’s preferred location.

**Table 1.** Baseline Characteristic of the Study Population (N = 11).

ID	Gender	Age^a^	Work Experience^a^	Profession	Oncological Discipline	Interview	Observation
P1	Female	51-60	>30	Nurse practitioner	Surgical	Yes	Yes
P2	Female	51-60	>30	Nurse practitioner	Surgical	Yes	No
P3	Female	51-60	>30	Medical specialist	Surgical	Yes	No
P4	Female	60-70	>30	Medical specialist	Medical	Yes	Yes
P5	Male	51-60	21-30	Medical specialist	Surgical	Yes	No
P6	Male	51-60	21-30	Medical specialist	Surgical	No	Yes
P7	Female	41-50	>30	Specialized nurse	Medical	Yes	Yes
P8	Male	41-50	11-20	Medical specialist	Surgical	No	Yes
P9	Female	51-60	>30	Specialized nurse	Medical	No	Yes
P10	Female	30-40	5-10	Resident	Surgical	No	Yes
P11	Female	51-60	>30	Specialized nurse	Medical	Yes	No

^a^Quantified in years.

### Observation Characteristics

Observations (N = 13) were conducted comprising ten patients. During seven outpatient clinic consultations, loved ones were present. The following consultations were observed: follow-up consultations (N = 5), goals of care conversations (N = 4), a first outpatient consultation (N = 1), a post-medical discussion (N = 1), and a post-operative consultation (N = 1). Furthermore, one multi-professional consultation meeting was observed, involving physicians and nurse practitioners deliberating optimal tumor treatment for patients at different illness stages. The observations lasted between 2 and 195 min.

### Contextual Collaboration

To properly understand and interpret the findings below, a description of the local collaboration, derived from the interviews and observations, is provided. At this outpatient clinic, patients are assigned to a contact person, a nurse, or a nurse practitioner when the patient’s situation is deemed complex. During consultations with the physician, the contact person has a supportive role. Support is offered during the consultation and afterwards by answering questions and discuss uncertainties. The physician discuss issues related to the physical dimension, while the nurse practitioners and nurses also focus on concerns in the psychological and social dimensions. Nurses and nurse practitioners mentioned multidisciplinary consultation while physicians did not. Documentation of VWN differed per participant. Physicians documented VWN if this affected treatment preferences whereas nurse practitioners and nurses also documented psychological/social issues.

### Findings

**Figure 1.**
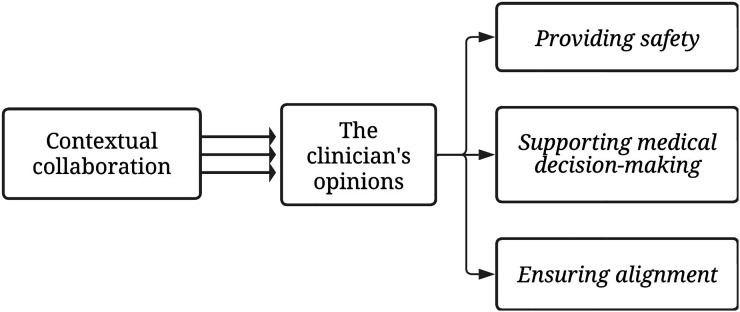
The exploration and monitoring of the patient’s VWN.

How participants’ explored and monitored the patient’s VWN emerged from their opinions rooted in the clinicians’ values and work experiences. These opinions influenced the attention paid to patients’ VWN. When and how attention was provided to patients’ VWN was shaped by the clinician involved. The data revealed differences between what participants mentioned during the interviews and what was observed during the observations. For example, most clinicians mentioned in interviews that they asked open-ended questions or broached specific topics with patients, such practices were largely not observed during the actual patient-clinician interactions. Instead, the observations revealed that clinicians tended to ask mostly closed questions. Three key opinions were identified: (1) providing safety, (2) supporting medical decision-making, and (3) ensuring alignment. Each opinion is given more detail below, illustrated with quotes. Figure 1 depicts the relatedness of the findings.

#### Opinion 1: Providing Safety

Participants indicated in interviews that they wanted to protect patients by supporting a sense of security and trust. *“That I know where the patient is in her life, what is important to her, what she still wants and what she no longer wants, and what she needs in terms of support”* (Interview P7). They described themselves as the patients’ protectors. Having a trusting relationship with the patient and knowing the patient’s background and medical situation were considered essential. *“I think that with us (nurses) it is more psycho-social. We find out more about the patient’s background and use that information in supporting them”* (Interview P7). To promote a sense of security, the role of contact person has been initiated. *“People do not always dare to ask the physician to repeat everything, but sometimes they still have some questions. So, I call them back and then we briefly check whether everything has been understood clearly”* (Interview P1). The observations revealed that the protection of the patient was reflected in the creation of a safe environment by ensuring time, attention, and empathy. In contrast, participants structured the provision of safety independently, generally not discussing their actions with the patient. “*The next appointment would be the last, I shortened it. I did that on purpose because I noticed that she is always very tense”* (Observation P6). Another aspect is reassurance that patients were always welcome to come back earlier or being accessible to patients if they had questions. *“I will be happy to look after you”* (Observation P5).

#### Opinion 2: Supporting Medical Decision-Making

Participants stressed the significance of providing information to inform and prepare patients, to enable decision-making and ensuring well-informed medical decisions. *“It is my role to verify that the patient has interpreted all the information correctly, so that she can make that decision properly”* (Interview P5). A few participants stressed verifying the alignment between decisions and the patient’s treatment preferences while considering the patient’s comprehension of the illness timeline. *“Make someone aware of the time frame they are in”* (Interview P4). Conversely, the observations varied among participants regarding the timing and methods used when certain matters were addressed, e.g., providing relevant information and important decisions. The information provided was based on participants’ perceptions of the patients’ decision needs. The utilization of Patient-Reported Outcome Measures (PROMs) was neither observed nor acknowledged by clinicians. Some participants emphasized treatment goal clarity, while others stressed the importance of instilling hope. *“That the patient feels that we are here for her. Sending her off with hope”* (Observation P4). Participants acknowledged their responsibility to inform patients, but obtaining and providing the desired information was not discussed regularly with the patient. *Patient: “I feel so sick from it”, “I have to face it again”, Physician: “Unfortunately, you have to face it again’, “I would do it”. “I would have liked it to be different for you”* (Observation P6).

#### Opinion 3: Ensuring Alignment

In interviews, participants considered alignment with patients about what should be discussed during a consultation and how this discussion was experienced important. In contradistinction, the observations revealed that, most participants aligned in an implicit manner by forming an image of the extent to which patients were ready to hear or discuss certain information. *“Normally, I always discuss sexuality, but I noticed that she found it a sensitive topic and that other people were present. I purposely did not discuss it”* (Observation P6). One participant referred to discussing complex subjects when judging a patient as resilient. *“I’m happy that you brought up this on your own because it is a difficult subject to discuss”* (Observation P4). A few participants focused on alignment by using open-ended questions. *“I will ask, ‘How did the conversation go, and do you want to talk about it?’”* (Interview P7). Patients were welcome to ask questions but, most often, time for questions was not provided. Some patients took the initiative to address what mattered to them.

## Discussion

This study aimed to gain insight into exploring and monitoring patients’ VWN with advanced cancer throughout the illness trajectory by hospital oncology clinicians. How clinicians explored and monitored patients’ VWN depended on their opinions originating from the clinicians’ personal values and work experiences. Three key opinions were identified: (1) providing safety, (2) supporting medical decision-making, and (3) ensuring alignment. These opinions were influenced by the hospital’s local collaboration. Exploring and monitoring VWN hinged on the clinician involved, with limited active alignment of care and treatment to patients’ VWN.

The significance of medical decision-making within the hospital setting is undeniable but decisions should be in line with patients’ VWN.^3^ The opinions of clinicians had impact on the attention for the patient’s VWN and decision-making. This corresponds with previous research that demonstrates the influence of clinicians’ values on decision-making by preselecting decision options and/or by influencing patients.^34,35^ Furthermore, in line with prior research, there was a discrepancy between what clinicians mentioned in interviews and what was observed in clinical practice.^36^ Clinicians appeared to overestimate their competence in communicating with patients and do not consistently follow through with their intentions.^37^ They aimed to facilitate decision-making by transferring medical information to patients but did not actively asked patients about their VWN. As a results, these VWN were not incorporated into the decision-making process. This may result in an insufficient understanding of the patient’s situation and priorities.^3,4^ While patients desire broader involvement in decision-making, extending beyond medical treatment to include aspects impacting their daily lives.^38,39^

### Strengths and Limitations

A strength of this study was combining qualitative methods for data collection, yielding rich data. Interviews and observations were continuously alternated, creating opportunities to further explore relevant findings.^27^ A limitation is that this study was conducted within one hospital. While our findings align with prior research, it is important to acknowledge that this may limit the transferability of our findings.^10^ Moreover, the study population was homogeneous in age and work experience, which might have influenced the way clinicians communicated with patients. Another limitation is that observations included patients with advanced cancer across a wide spectrum of illness trajectories, ranging from those undergoing intensive systemic and/or other treatments with curative intent to those receiving palliative treatments. However, communication about VWN should begin early in the illness trajectory, regardless of whether patients are receiving treatment with curative or palliative intent. Additionally, the majority of participants were female (73%), which aligns with national figures indicating that 70% of employees in Dutch academic hospitals are female ^40^ Hence, we expect our results to align with the current clinical practices and that inclusion of mostly female participants and patients treated with curative intent (N = 4) possibly did not affect the trustworthiness of the results.^38,41^

### Implications for Clinical Practice and Recommendations

Presently, as a patient, the timing and way attention is provided to your VWN within the hospital setting, depends on the clinicians you may encounter. PROMs, which are proven suitable for mapping multidimensional wishes and needs, are helpful to optimize systematic attention for the patient VWN.^42^ Its use fosters a comprehensive understanding of patients’ situations, their priorities and facilitates communication.^42^ This supports clinicians not only to transfer medical information to patients but also systematically assess and discuss what matters to them, thus engaging them more in communication and decision-making. The Utrecht Symptom Diary – 4 Dimensional (USD-4D) is a PROM that can be used to explore and discuss symptoms and issues in the psychical, psychological, social, and spiritual dimensions during the illness trajectory.^43^ When integrating PROMs in clinical practice, engaging clinicians with expertise in palliative care is advisable as they can provide support in informing patients and guiding appropriate decision-making.^44^ This involvement may serve as informal training-on-the-job since communication competences are not acquired solely through formal education but partially through observation and modelling others in clinical practice.^45,46^ Finally, the findings of this study underscore the significance of promoting clinicians’ awareness of their own opinions and the influence these opinions have on the provision of care. A professional learning culture that encourages and supports clinicians in reflecting on the importance of appropriate care seems crucial.^47^ This reflection, facilitated through self-reflection and peer feedback, appears essential for optimizing systematic attention to the VWN of the patient.

### Implications for Future Research

It is unknown how patients with advanced cancer experience the attention for their VWN throughout the illness trajectory. Insight into patients’ perspectives is essential since patient-reported experiences are key to improving the quality of care.^48^ Future qualitative research from a patient perspective is therefore recommended to map their expectations and experiences.

## Conclusion

While clinicians recognized the importance of exploring and monitoring patients’ VWN, a systematic approach was lacking, resulting in varying practices. Three key opinions influencing individual practices were identified: providing safety, supporting medical decision-making, and ensuring alignment. Given that appropriate palliative care is based on patients’ VWN, should patients’ be actively engaged in communication and decision-making rather than primarily providing them with medical information. Enhancing the integration of PROMs to facilitate communication and ensure continuity of individual practices, supported by a professional learning culture could improve appropriate palliative care in hospital cancer care.

## Data Availability

All data pertaining to this study are managed in accordance with legal and organizational guidelines. Transcripts and field notes will not be released publicly to safeguard confidentiality and anonymity. The coding tree is available from the corresponding author upon reasonable request.*

## Appendix 1: Topic Guide for the Interviews

**Table:**

- The topics discussed when attention is given to the patient’s VWN
- The moments when attention is given to the patient’s VWN.
- Starting a conversation about the patient’s VWN
- Exploring the patient’s VWN
- Monitoring the patient’s VWN
- Collaboration and consultations with colleagues about the patient’s VWN
- Documentation of the patient’s VWN

## Appendix 2: Observation Framework

Contextual information

**Table:**

1. Place	The physical place
2. Actor	The people involved
3. Activity	A series of related actions that people do
4. Object	Physical items that are present
5. Action	Individual deeds carried out by people
6. Event	A series of related activities that people do
7. Time	The sequence of something with regard to time
8. Aim	The objectives that people try to achieve
9. Feeling	The emotions people feel and express

Conversation description^21^

**Table:**

Six Function Model of Medical Communication	Goals	Immediate Endpoints	Intermediate and/or Surrogate Endpoints	Long Term Endpoints
1. Fostering the relationship	Good and effective relationship	+ Eye contact	+ Trust	+ Patient satisfaction
		+ Patient participation	+ Sense of rapport	+ Patient health
		- Physiological stress measurement	+ Satisfaction with consultation	- Physician stress and burn out
2. Gathering information	Adequate diagnosis and/or interpretation of symptoms	+ Explorative behavior	+ Adequate diagnosis/treatment plan	+ Patient health
		+ Expression of patients concerns	- Diagnostic test ordering	+ Physician satisfaction
			- Medical errors	
3. Information provision	Good information provision	+ Check understanding/explore prior knowledge	+ Recall	- Patient uncertainty
		- Use of jargon	+ Understanding	+ Patient autonomy
4. Decision-making	Decision based on information and preferences	+ Check decision-making preference/patient values	- Decision conflict	+ Satisfaction with decision
		+ Provide information	+ Satisfaction with decision	+ Patient health
5. Enabling disease and treatment-related behavior	Adequate and feasible f and treatment-related behavior	+ Address patient motivation and efficacy	+ Illness-related behavior	+ Patient health
			+ Treatment adherence	
			+ Lifestyle	
			- Costs	
6. Responding to emotions	Support the patient, enhancing the communication and referral where needed	+ Clinician explorative skills/silence	+ Patient since receiving support	+ Patient emotional adjustment
		+ Patient expression of emotions	+ Treatment of psychopathology	- Psychological distress
		- Time constraints		- Costs
